# Adipose and Bone Marrow Derived-Mesenchymal Stromal Cells Express Similar Tenogenic Expression Levels when Subjected to Mechanical Uniaxial Stretching *In Vitro*

**DOI:** 10.1155/2023/4907230

**Published:** 2023-01-30

**Authors:** Hui Yin Nam, Mohd Rusdi Draman @ Yusof, Tunku Kamarul

**Affiliations:** ^1^Tissue Engineering Group, Department of Orthopaedic Surgery (NOCERAL), Faculty of Medicine, Universiti Malaya, 50603 Kuala Lumpur, Malaysia; ^2^Nanotechnology and Catalysis Research Centre (NANOCAT), Universiti Malaya, 50603 Kuala Lumpur, Malaysia; ^3^Advanced Medical and Dental Institute (AMDI), Universiti Sains Malaysia, Bertam 13200 Kepala Batas, Pulau Pinang, Malaysia

## Abstract

The present study was conducted to determine whether adipose derived mesenchymal stromal cells (AD-MSCs) or bone marrow derived-MSCs (BM-MSCs) would provide superior tenogenic expressions when subjected to cyclical tensile loading. The results for this would indicate the best choice of MSCs source to be used for cell-based tendon repair strategies. Both AD-MSCs and BM-MSCs were obtained from ten adult donors (*N* = 10) and cultured *in vitro*. At passaged-2, cells from both groups were subjected to cyclical stretching at 1 Hz and 8% of strain. Cellular morphology, orientation, proliferation rate, protein, and gene expression levels were compared at 0, 24, and 48 hours of stretching. In both groups, mechanical stretching results in similar morphological changes, and the redirection of cell alignment is perpendicular to the direction of stretching. Loading at 8% strain did not significantly increase proliferation rates but caused an increase in total collagen expression and tenogenic gene expression levels. In both groups, these levels demonstrated no significant differences suggesting that in a similar loading environment, both cell types possess similar tenogenic potential. In conclusion, AD-MSCs and BM-MSCs both demonstrate similar tenogenic phenotypic and gene expression levels when subjected to cyclic tensile loading at 1 Hz and 8% strain, thus, suggesting that the use of either cell source may be suitable for tendon repair.

## 1. Introduction

Mechanical forces are known to play a fundamental role in cell behaviour and their adaptation to their environment. These are particularly essential to maintain tissue homeostasis [1]. In tendons, it has been demonstrated that stretching produces superior repair outcomes [2–4], whereas disuse atrophy and tissue degeneration will occur when these tissues are not mechanically loaded for a long period of time [5, 6]. These have shown to result in the complete loss of tissue function [7]. It has become apparent that mechanical cues play a vital role in cellular signalling, through a series of molecular interactions, that results in genetic and protein expressions that maintain cellular function [8, 9]. Using this knowledge, a number of studies have attempted to exploit this process, known as mechanotransduction, to manipulate cell fate and outcomes [10–14]. One such attempt has included the use of mechanical stimuli in regulating the cellular differentiation of multipotent progenitor cells. The use of human mesenchymal stromal cells (MSCs) has garnered strong interest due to its ability to undergo self-renewable and multilineage differentiation. As such, MSCs have been sought after as a potential cell source to improve tissue repair and regeneration since these cells also exhibit prohealing and immunomodulatory effects [15–17]. Indeed, one method by which these cells can promote tissue repair is the differentiation of these cells that are implanted into damaged sites, as proposed by other researchers [18, 19]. Once MSCs are nested within the matrix of the target site, these cells will differentiate towards the native resident cells thereby producing the necessary protein that would promote local tissue healing [20, 21]. This process can be regulated through several pathways such as local cytokine signalling, cell-surface interaction, and local tissue mechanical impulses [22]. From our own experience, intrainjection of autologous MSCs in joints with damaged cartilage results in moderate to good repair outcomes due to the presence of these cells within the damaged sites [23]. There has been a study indicating that 24% of injected MSCs were retained at the site of injury after 24 hours [24], and that these cells may undergo differentiation over time. The *in vivo* differentiation could be further enhanced when mechanical loading is then performed in the form of exercise regime [25]. In an attempt to further understand the underlying mechanisms that promote cellular differentiation, cells seeded in scaffolds and were subjected to mechanical loading *in vitro* [26], and the study shows that the loaded construct demonstrates differentiation expression, thus suggesting that mechanical loading may be the main contributor to the cell differentiation process.

In our previous studies, we were also able to demonstrate that by subjecting MSCs to cyclical stretching, these cells would undergo cellular differentiation and produce superior tendon protein expression [27, 28]. Such changes have also been observed in chondrocytes subjected to compressive loading in other studies [29, 30]. Together with our previous observation of cellular differentiation in damaged sites when cells are injected into these areas, we can therefore suggest that the mechanotransduction process that occurs in the transplanted MSCs may have been responsible for the observed positive outcomes [31]. Whilst the direct evidence of the direct role of mechanotransduction remains debatable, what cannot be refuted is the fact that MSCs do provide a significant positive musculoskeletal tissue repair outcome.

It is previously demonstrated that bone marrow has been a traditional source for MSCs harvest since bone marrow derived mesenchymal stromal cells (BM-MSCs) have demonstrated predictable results in musculoskeletal tissue engineering. There is a drawback in using BM-MSCs mainly due to invasiveness of the procedure involved in extracting these cells. It has been reported regularly that extraction from bone marrow results in minor donor site morbidity [32]. This, in addition to the low yield of cells from the bone marrow stroma [33], results in many studies describing alternative sources for MSCs. One such source is from adipose tissues. Adipose tissue derived mesenchymal stromal cells (AD-MSCs) have been shown to contain an abundance of MSCs, and since subcutaneous fat deposits are in large quantity in the human body, extracting these cells are easier and less painful for the donor [34, 35]. Despite its many promises, the potential use of AD-MSCs for the repair of damaged tendon does not appear to be explored with presently no previous works describing its potential when subjected to mechanical stimulation, i.e., stretching. Furthermore, while there has been some progress made in better understanding on how mechanical signals are sensed by MSCs [36, 37], the mechanotransduction processes that occur during tensile loading have not been well described.

Therefore, to demonstrate the potential efficacy of AD-MSCs as a source for tendon regeneration, a study was conducted to determine the tenogenic expression potential of these cells when subjected to cyclic tensile loading. This will be compared to BM-MSCs, which is presently being used as a therapeutic cell source for damaged tendons. It is hoped that the study will be able to demonstrate as to whether AD-MSCs can indeed be considered as a good candidate for tendon repair, and that further studies to develop this cell source should be conducted.

## 2. Materials and Methods

### 2.1. Bone Marrow and Adipose Tissue Harvesting from Patients

Five grams of adipose tissue sample and 5 cc of bone marrow were collected each from donors (*N* = 10; for MSCs isolation purpose) (mean age = 65.3; 6 females and 4 males) undergoing orthopaedic-related surgeries in University Malaya Medical Center with approval from the Medical Ethics Committee of University Malaya Medical Center (reference number: 20149-563). These samples were kept on ice throughout the transportation to the laboratory and processed for MSCs isolation within few hours after samples harvesting. From the 10 donors, 4 of the donors were used for quantitative experiments including cell proliferation assessment, total collagen assay, and gene expression assay, while the other 6 donors were used for qualitative/semiqualitative experiments including morphology assessment and MSCs characterization.

### 2.2. Isolation and Culture of Human Adipose Derived-MSCs (AD-MSCs)

**Figure 1:**
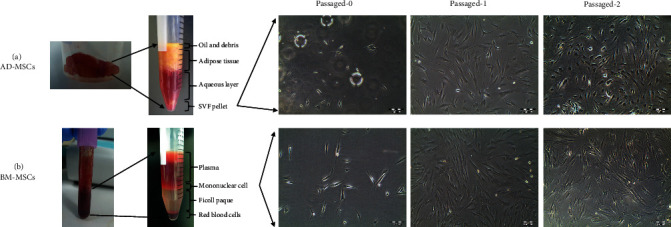
Morphology of isolated human AD-MSCs and BM-MSCs from adipose fat pad and bone marrow, respectively. (a) Enzymatic digestion of adipose tissue using collagenase type I, and the MSCs population isolated from the SVF. (b) MSCs isolated from bone marrow and the mononuclear cells isolated by Ficoll density centrifugation. At primary culture passage-0 (Day 6), fibroblastic as well as small clear cells can be observed. The number of clear cells was reduced during the passages and fibroblastic cells appeared to be the dominant cell type.

To isolate AD-MSCs, the harvested adipose tissue sample was rinsed using 1X phosphate-buffered (PBS) saline containing 1% penicillin-streptomycin until all visible blood and excessive fluid were eliminated. Small vessels and unwanted tissues were dissected away from the sample. Subsequently, the mixture was added with 0.1% (v/w) type I collagenase and incubated at 37°C for 1 h to allow the enzymatic digestion process to occur. After centrifugation, the pellet at the bottom of the tube containing the stromal vascular fraction (SVF) was collected (Figure 1) and transferred a cell culture flask containing complete cell growth medium (low-glucose DMEM supplemented with 10% fetal bovine serum, 1% of penicillin-streptomycin, and 1% GlutaMAX™-I) (Invitrogen-Gibco, USA) and incubated at 37°C in a humidified 5% CO_2_ incubator. After 4 days of culturing, the digested tissue was then removed from the cell culture flask and discarded completely. Culture medium was changed every 3 days until reaching 80-85% of cell confluency. The AD-MSCs were subcultured up to passage 2 to be used in our experiments.

### 2.3. Isolation and Culture of Human Bone Marrow-Derived-MSCs (BM-MSCs)

To isolate BM-MSCs, cell isolation was performed using our standard laboratory protocol as described in our previous publication [38, 39]. Briefly, bone marrow specimen was diluted with 1X PBS and gently layered onto the top of the density of 1.077 g/mL Ficoll-paque solution (Amersham Biosciences, Sweden). The mononuclear cell layer was collected after undergoing gradient density centrifugation at 2,200 rpm for 25 min (Figure 1). The cell pellet was then extracted after second centrifugation and plated on a tissue culture flask containing complete cell growth medium. The cells were maintained in a humidified incubator at 37°C with 5% CO_2_ on air. The subsequent medium changes were conducted at 3 day-intervals until 80%-85% confluence was reached. Cells were serially passaged until passage-2 prior to further experiments.

### 2.4. Characterization of Human AD-MSCs and BM-MSCs

To determine whether cells obtained were hMSCs, various tests including flow cytometry analysis for specific cell surface markers, cell morphological images, and the ability of the isolated cells to undergo trilineage differentiation were conducted (cells from 6 donors).

#### 2.4.1. Cellular Morphology

Serial microscopic examinations were carried out at 3-day intervals in order to assess physical characteristics of the cells. The cellular morphology of cultured AD-MSCs and BM-MSCs at passage 0 (P0), passage 1 (P1), and passage 2 (P2) were captured using an inverted phase contrast microscope (Olympus CKX 41, Japan).

#### 2.4.2. In Vitro Trilineage Differentiation

The multipotent capability of AD-MSCs and BM-MSCs were tested using specific StemPro® differentiation supplements (Invitrogen-Gibco, USA), inducing the cells into adipogenic, osteogenic, and chondrogenic lineages, with triplicates for each lineage, as described in our previous established protocol [28, 39]. The differentiation of these cells was confirmed through their phenotypic expression. The confluent passaged-3 cells were cultured with differentiation medium, respectively. The differentiation medium was changed every 3 days.

Briefly, for the adipogenic differentiation, 14 days after the culture initiation the cells were fixed with 4% paraformaldehyde for 30 min, rinsed with 60% isopropanol, and stained with Oil Red O (Sigma-Aldrich, USA) for 10 min. The slides were kept wet to keep the lipid vacuoles from disrupting. The slides were viewed and captured using light microscope (Nikon Eclipse TE2000-S, Japan). For osteogenic differentiation, 21 days after culture initiation, the filtered 2% Alizarin Red solution (pH 4.2) was added to the fixed cells for 3 min. Alizarin red staining was used to observe the matrix mineralization. To induce chondrogenic differentiation, pellet culture system was used. Twenty-eight days after the initiation of the culture, each chondrogenic cell pellet (1 × 10^6^ passaged-3 cells) was fixed in 10% neutral buffered formalin for 1 hour and went through tissue processing (dehydrating in ascending concentrations of ethanol and clearing in xylene) overnight. The sample was then embedded in paraffin wax and sectioning at 4 *μ*m using a microtome. The sections were then stained with 0.1% aqueous Safranin O for 5 min.

#### 2.4.3. Evaluation of Cell Surface Markers by Flow Cytometry

Human AD-MSCs and BM-MSCs (1 × 10^6^ cells/mL) were trypsinized, and the cells were washed with 1X PBS and resuspended in 100 *μ*L of FASC stain buffer (BD Biosciences, CA, USA) before being transferred into polystyrene round-bottomed tube. Fluorescein isothiocyanate (FITC), or phycoerythrin (PE), or peridinin chlorophyll protein (PerCPCY5.5), or allophycocyanin-(APC-) conjugated antimarker mAbs were used to stain the cells for 15 min in the dark. The tested markers including CD44, CD73, CD90, CD105, CD14, CD34, CD45, and HLA-DR were tested [38, 39]. After incubation, the cells were washed and then analysed using a flow cytometer (BD FACS Cantor II, BD Biosciences, CA, USA) with FACS DIVA software (BD, NJ, USA). Unstained and/or matched isotype controls were used to set background fluorescence levels.

### 2.5. Cell Seeding and Mechanical Straining System Set up

A total of 0.02% collagen type I (Sigma, St. Louis, USA) was used to coat the autoclaved transparent elastic silicone chambers (Strex, Japan). A total of 1 × 10^4^ cells/cm^2^ AD-MSCs and BM-MSCs were seeded into each silicone chamber, respectively. After 48 h of culture, the medium was replaced with medium containing 1% FBS for 24 h. This step was conducted in order to synchronize the condition (by arresting the cells at the G0/G1 stage of their cell cycle progression) at the beginning of each experiment. Following 24 h of synchronization, the cell culture medium with a standard growth medium containing 10% FBS with no additional differentiation growth factors was replaced prior to mechanical stretching. The silicone chambers were mounted on a mechanical stretch device (ST-140-10, Strex, Cupertino, USA). A stretching rate of 1 Hz and a strain of 8% were applied to the AD-MSCs and BM-MSC seeded silicone flasks. The specimens were collected at 24 h and 48 h. Unstrained cells on silicone chambers in the same culture environment were used as control for this study.

### 2.6. Microscopic Evaluation

Microscopic images of the experimental cells at each time point were captured using an inverted tissue culture CCD camera-assisted microscope (Olympus CKX 41, Japan). Images from four visual fields of the cells were randomly captured. The morphology and alignment of the unstrained and strained cells on elastomeric substrate were then compared.

### 2.7. Cell Proliferation Assay

The alamarBlue® (AB) assay was used to assess cell proliferation. This assay utilizes the colorimetric quantitative analytical principle. At 0, 24, and 48 h, 10% AB reagent was added to the unstrained and strained cells in the culture medium. The samples were then incubated for 4 h at 37°C in a cell culture incubator, protected from direct light exposure. A total of 100 *μ*L of the alamar-containing medium was collected and transferred to a 96-wells plate. The absorbance measurement was read on a microtiter plate reader at 570 nm wavelength while using 600 nm as a reference wavelength. Following the manufacturer's protocol, the percentage of AB reduction was calculated. For background values, medium without cells was used as the negative control group to correct the values of % AB reduction.

### 2.8. Total Collagen Biochemical Assay

A Sircol™ collagen assay kit (Biocolor, UK) was used to measure total extracellular soluble collagen. At 24 and 48 h of the experiment, the culture medium was collected from the experimental cells and mixed with 1 mL of Sircol dye reagent with vigorous agitation for 30 min. The mixtures were then centrifuged at 12,000 rpm for 10 min to collect the collagen dye complex. The unbound dye solutions were removed by draining the tubes carefully and washed the dye with ice-cold acid-salt wash reagent by centrifugation at 12,000 rpm for 10 min. The dye (which was bounded to the collagen pellet) was solubilized by adding 1 mL of alkali reagent. The absorbance of the samples was measured at 555 nm wavelength.

### 2.9. Gene Expression Assay

Total RNA from unstrained and strained cells was extracted using the RNeasy mini kit (Qiagen, USA) according to the manufacturer's instructions. RNA concentration and purity were determined using spectrophotometer (Nano-Photometer, Germany) at the setting of A260/280. RNA integrity was verified by visualizing 18S and 28S rRNA bands on formaldehyde-agarose gels. Only samples with good quality were selected for RT^2^ Profiler PCR arrays downstream analysis. An equal amount of RNA (500 ng) was used for reverse transcription using the RT^2^ First Strand Kit (Qiagen, USA) using protocol steps that eliminated genomic DNA. qPCR experiments were performed using the customised RT^2^ Profiler PCR array (Cat No./ID: CLAH22023) (SABioscience, USA) and RT^2^ SYBR Green qPCR Mastermix (Qiagen, USA) on a real-time PCR instrument (CFX96, BioRad, USA). The temperature protocol included a start cycle for 10 min at 95°C, 40 cycles of amplification (15 s at 95°C and 15 s at 60°C), followed by a melt curve. The PCR array profiles the expression of selected nine genes (Table 1) involved in mesenchyme lineage. The housekeeping gene were *PGK1* (phosphoglycerate kinase 1) and *HPRT1* (hypoxanthine phosphoribosyltransferase 1). The housekeeping genes, RT controls, and PCR controls were included in each run. Relative expression of target genes was determined using the ΔΔCq method where the unstrained cell is the control group.

### 2.10. Statistical Analysis

The assays (cell proliferation, total collagen biochemical, and gene expression) were carried out in technical triplicates (*n*) per experimental run, using four independent samples from different donors (*N*) for each group. The data is presented as mean ± standard deviation (SD). Student's *t*-test was carried out to compare the differences in mean values. Statistical analyses were performed using SPSS software version 15.0 (SPSS Inc, USA), which took a probability value of *p* < 0.05 as statistically significant.

## 3. Results

### 3.1. Characterization of Human AD-MSCs and BM-MSCs

Results revealed that the morphology, surface antigen expression profiles, and the multidifferentiation capacity of human AD-MSCs and BM-MSCs were similar and conformed to the International Society for Cellular Therapy (ISCT) minimal characteristics of MSCs [40].

#### 3.1.1. Plastic-Adherent Fibroblast-like Cells

After going through the culturing process in standard growth medium at 37°C incubation for 24 h, a proportion of the isolated cells from both adipose tissue and bone marrow demonstrated adherence to the plastic flask surface. Following medium change after 5 days of culture, they aggregated to form colony-forming-units. Rounded cells were observed to change into fibroblast-like morphology where the cells appeared spindle-shaped. Their appearance, however, varied, with heterogeneous shapes were observed, with features of elongated cells and multipolar projections. After 2 weeks in culture, the cells started reaching confluency and demonstrated fingerprint-like orientation. The cells appeared more homogeneous after cell passaging. Both AD-MSCs and BM-MSCs at passage-2 exhibited spindle-shaped morphology. However, AD-MSCs apparently grew at a relatively higher rapid rate compared with BM-MSCs, which could be observed under light microscopy (Figure 1).

#### 3.1.2. In Vitro Trilineage Differentiation

**Figure 2:**
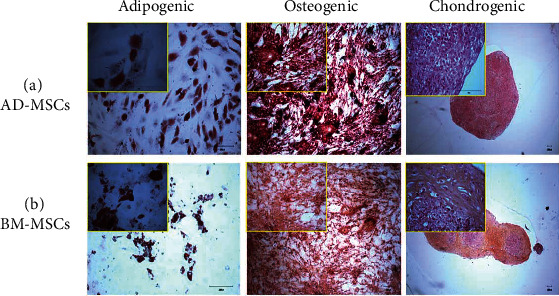
Trilineage differentiation potential of primary for (a) AD-MSCs and (b) BM-MSCs in adipogenic differentiation, osteogenic differentiation, and chondrogenic differentiation.

Both AD-MSCs and BM-MSCs showed positive results for the differentiation experiments (Figure 2). This indicated that the MSCs isolated from the two cell types had multipotent ability, having the capacity to undergo trilineage differentiation which included osteogenic, adipogenic, and chondrogenic mesodermal lineages.

In adipogenic culture conditions, small lipid droplets appeared in both cell types on day 3, and they gradually spread homogenously. Not surprisingly, AD-MSCs displayed highly adipogenic cells with abundant Oil Red O positive lipid droplets as compared to BM-MSCs. This indicated that adipocyte formation was more extensive in AD-MSCs. Lipid vacuoles were not present in control cultures of either AD-MSCs or BM-MSCs. In osteogenic culture, nodule-like structures were observed in certain regions. By using Alizarin Red S, both cell types appeared red showing the presence of mineral deposition. In comparison, no accumulation of calcium oxalate crystals was observed in noninduced control MSC cultures that were stained. In the pellet culture system for chondrogenic differentiation, the size of the pelleted cells, i.e., AD-MSCs and BM-MSCs seemed to be increasing over time. Using Safranin O, the matrix of the both cell types demonstrated a pink-red colour, indicating sulphated proteoglycans or glycosaminoglycan deposition.

#### 3.1.3. Immunophenotype Expression

**Figure 3:**
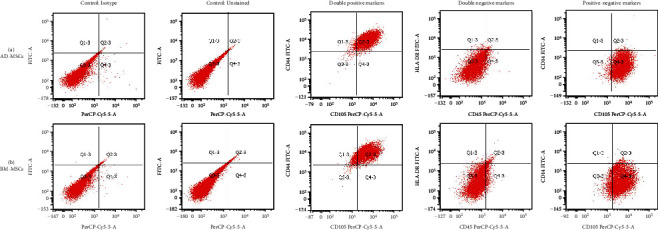
Immunophenotypic characterization of the surface of (a) AD-MSCs and (b) BM-MSCs using flow cytometry. The representative images showed the both type of MSCs expressed at least 85% of double-positive expression, double-negative, or coexpressed its positive and negative markers.

Cells derived from adipose tissue and bone marrow expressed positive surface markers for CD73, CD44, CD90, and CD105 (Figure 3), which are markers for hMSCs (Table 2), particularly highly expressed were CD44 and CD90. They were negative for CD14, CD34, CD45, and HLA-DR marker expression. This indicated that the cells were not of hematopoietic or leucocytic in origin.

### 3.2. Effects of Cyclic Mechanical Stretch on Cell Morphology and Alignment

**Figure 4:**
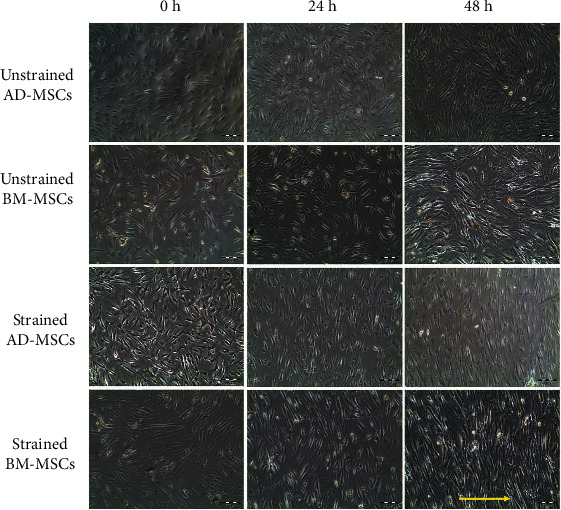
Effects of cyclic tensile loading on the morphology and orientation of both AD-MSCs and BM-MSCs. The strained cells presented an orientation perpendicular to the strain axis. The substrate was stretched in the direction of the yellow arrow.

To determine the effects of mechanical stretch on cell morphology and organization, uniaxial cyclical tensile loading at 1 Hz of frequency and 8% of strain was applied on AD-MSCs and BM-MSCs. Mechanical stretch markedly altered the morphology and alignment of cells. The MSCs were randomly oriented before mechanical stimuli was applied (0 h), while both the AD-MSCs and BM-MSCs appeared to be perpendicular orientated to the direction of stretching after exposure to cyclic stretching. It also appears that the changes correspond to the duration of stretching (Figure 4). This may be due to the adaptation process of the cells, where cellular tensegrity tends to minimise the shape in order to reduce stresses in response to mechanical forces. In contrast, both unstrained AD-MSCs and BM-MSCs groups showed no specific cellular orientation, similar with 0 h group. The number of cells in all strained and unstrained groups appeared to increase with time. The increase in the number of cells was seen clearly especially for unstrained cells at 48 h group compared to the 0 h group. However, changes of cell number appeared small in strained cells compared to unstrained cells. Cells when strained exhibited a different morphology than unstrained cells, where the strained cells appeared elongated. There was no difference in morphology between the AD-MSCs and BM-MSCs strained group, where both cell types demonstrated similar appearance of spindle-shaped cells.

### 3.3. Effects of Cyclic Mechanical Stretch on Cellular Proliferation

**Figure 5:**
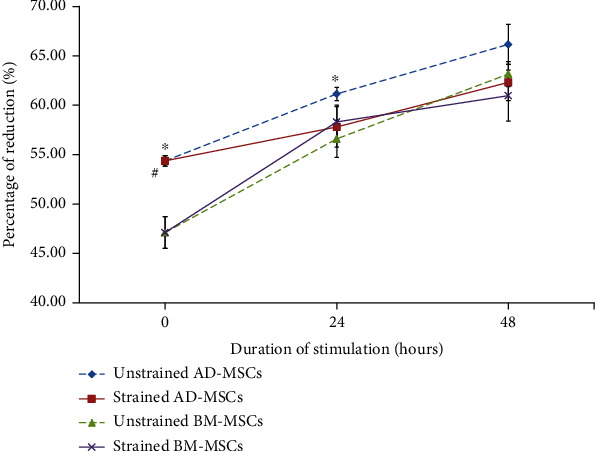
Comparison between the cellular proliferation of AD-MSCs and BM-MSCs with or without mechanical stimulation at different durations of cell culture. The cell proliferation rate higher in AD-MSCs as compared to BM-MSCs when left to grow on silicone chambers. This did not appear to be the case when cells were subjected to cyclical stretching at 8% and 1 Hz. There was no significant difference for both these types of cells and when compared to unstrained cells when mechanical stimulation was applied. Significance (*p* < 0.05) was indicated with an asterisk (∗) which compared unstrained AD-MSCs and unstrained BM-MSCs in different duration, while significance (*p* < 0.05) was indicated with a hash (#) which comparison between strained AD-MSCs and strained BM-MSCs in different duration. *N* = 4, *n* = 3. Error bar = ±SD.

The cellular proliferation rate of AD-MSCs and BM-MSCs was compared using alamarBlue absorbance reduction. Overall, alamarBlue® assay revealed that both unstrained and strained threads support the growth of human MSCs.  Figure 5 shows that in unstrained conditions, AD-MSCs have higher cell proliferation compared with BM-MSCs, with statistically significant difference observed at 0 h and 24 h. Both cell types demonstrated gradual proliferation over time. However, when the MSCs were subjected to stretching, there was no difference between AD-MSCs and BM-MSCs either at the 24 h or 48 h time points. Mechanical stretching at 1 Hz and 8% demonstrated not to enhance cell proliferation in either cell group especially in AD-MSCs. In contrast, BM-MSCs were enhanced by mechanical stimulation after 24 hours of stretching, although it was not statistically significant. Nevertheless, a trend towards a decrease in cell proliferation was observed after 48 hours of stretching. These results suggest that the proliferation rate of human MSCs is not influenced by stretching at 1 Hz and 8%, regardless whether MSCs derived from adipose tissue or bone marrow.

### 3.4. Effects of Mechanical Stretch on Total Collagen Expression of MSCs

**Figure 6:**
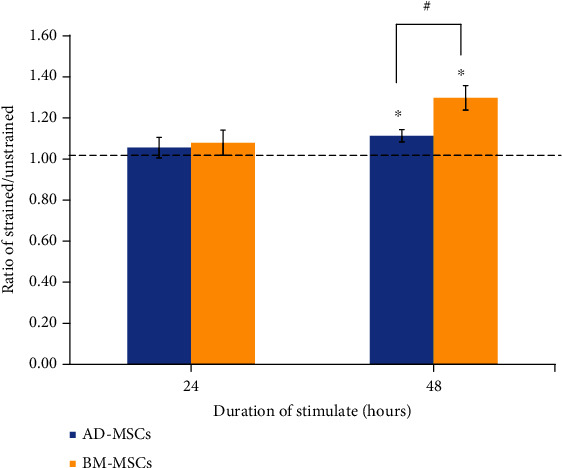
Extracellular collagen content analysis of AD-MSCs and BM-MSCs cultured at 1 Hz+8% at different duration of stretching. Statistical significance (*p* < 0.05) was represented by an asterisk (∗) which was compared to the unstrained cells represented by the Y axis (indicated as 1.00). Statistical significance (*p* < 0.05) was also represented by a hash (#) which is a comparison between AD-MSCs and BM-MSCs. *N* = 4, *n* = 3. Error bar = ±SD.

The results showed that uniaxial stretching increased collagen production in cell cultures (Figure 6). At 1 Hz and 8% strain, when compared with unstrained groups with a normalized value 1, an increase in total collagen was noted in both AD-MSCs and BM-MSCs at both 24 h and 48 h. However, the increase of the collagen production was only statistically significant (*p* < 0.05) at 48 h. In terms of comparison between the AD-MSC and BM-MSC group, the BM-MSC group produced more collagen than the AD-MSC group at both time points, but only significantly different at 48 h.

### 3.5. Effects of Cyclic Mechanical Stretching on Mesenchyme Differentiation of MSCs

**Figure 7:**
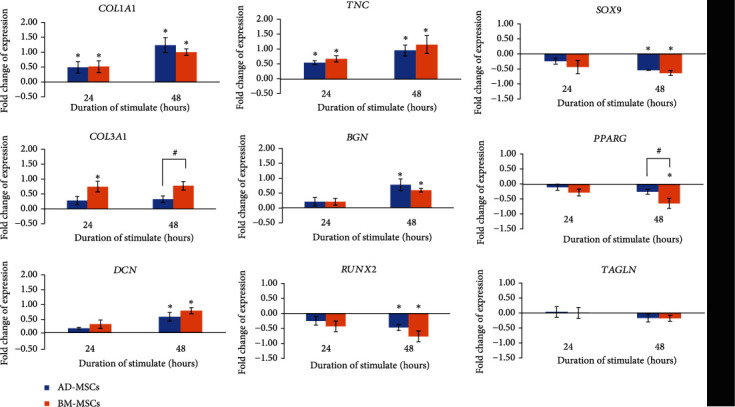
mRNA expression level of different genes in AD-MSCs and BM-MSCs subjected stretching at 24 h and 48 h. The expression level of each gene was normalised with the level of average housekeeping genes. The value of fold change was presented as ratio of strained group to the unstrained group. Statistical significance (*p* < 0.05) was represented by an asterisk (∗) in comparison to the unstrained cells represented by the Y axis (indicated as 0.00). Statistical significance (*p* < 0.05) was also represented by a hash (#) which is a comparison between AD-MSCs and BM-MSCs. *N* = 4, *n* = 3. Error bar = ±SD.

To investigate the regulatory genes during the tenogenic process when cells are subjected to cyclic tensile loading, the mRNA level of *COL1*, *COL3*, *DCN*, *TNC*, and *BGN* were determined. Both AD-MSCs and BM-MSCs (Figure 7) were triggered by mechanical simulation towards tenogenic lineage, but not to other mesenchyme lineages including osteogenic (*RUNX2*), chondrogenic (*SOX9*), adipogenic (*PPARG*), and smooth muscle (*TAGLN*). Instead, our investigation demonstrated a downregulation of expression levels. Uniaxial strain regulated matrix remodeling by increasing *COL1* and *COL3* expression. The level of *COL3* expressed was higher in BM-MSCs than AD-MSCs. Compared to the collagen group, *DCN* also showed a similar pattern, where the expression was higher for a longer duration of stretching. The results showed *TNC* and *BGN* expression were upregulated in the both cells types, more notably at 48 h. However, after stretching, the BM-MSCs showed higher and faster tenogenic gene expression than AD-MSCs although not significantly. These results suggest that both AD-MSCs and BM-MSCs have good potential to undergo tenogenic differentiation through mechanical stimulation.

## 4. Discussion

The present study demonstrated that from the same donor, no obvious differences can be observed between the morphology and response towards mechanical stretching in both AD-MSCs and BM-MSCs. These were also apparent in our flow cytometry, morphometric analysis, and characterization analysis. Although generally similar, there were some minor differences such as CD34, a surface marker of hematopoietic cells, which appeared to be slightly higher expressed in AD-MSCs, i.e., 5.5%. This, however, is not unexpected since such findings were also reported to be present up to 8.23% of the cell population [41]. It was demonstrated that the expression of CD34 in freshly isolated adipose stem cells will reduce over several passages but will retain some of its expression and not always completely absent [42]. When investigations were made to determine their proliferation and reorientation ability subjected to with or without stretching, both cells demonstrated contrasting outcomes. AD-MSCs proliferated better in static cultures. This finding is similar to a previously reported study where cell doubling time of AD-MSCs is 2 days as opposed to 7 days in BM-MSCs [41]. But when cyclic loading was applied, there were no differences observed. This observation is not unexpected, since we have demonstrated that lower strain values produced higher proliferation rates [38]. In this study, uniaxial cyclic strain modality is selected over other mechanical strain methods as it is thought to better mimic the type of mechanical strain experienced by MSCs in the human body [43]. What is worth noting is that cyclic loading results in the change in cellular proliferation rates to both MSCs types, demonstrating similar levels. This suggests that the internal mechanisms regulating cellular proliferation are stretch sensitive and may reset or overcome the natural cellular proliferation programming that exists in static culture conditions. This, however, would need to be proven in a more robust experiment.

Similar to *in vivo* conditions, both AD-MSCs and BM-MSCs are mechanosensitive and will realign in an arrangement perpendicular to the direction of loading. These changes were also time dependent and produced more prominent reorientation patterns over time. It has been suggested that when subjected to cyclic loading, actomyosin fibres undergo stretching that threatens cellular tensegrity [44]. The cell will strive to survive by elongating its shape and thus minimizing the energy required to maintain its integrity. The change in cell alignment and of adaptive processes through morphological changes are of natural physiological response and have resulted in the reorganization of cells' axes close to 100-110 degrees from the direction of loading [45, 46]. This in turn would avoid cell detachment or cell anoikis, which ultimately can lead to cell death [47, 48]. From our own experience, we were able to demonstrate that uniaxial tensile strain can significantly increase the Young's Modulus of the cell using cyclic loading modality, owing to the increased alignment of cytoskeleton components including F-actin fibres, thereby reducing the chance of cell failure [28].

In terms of their differentiation potential, both the protein and gene expression analyses demonstrated a distinctive cellular differentiation towards tenogenic differentiation that of any other mesenchymal lineage. Indeed, between the two, BM-MSCs produced higher total collagen protein and *Col3* gene expression than AD-MSCs at 48 hours, signifying an increase of selected tenogenic expression rather than a global tenogenic expression. It has been previously suggested that appropriately managed mechanical loads at physiological levels would positively influence the expression of ECM and therefore stimulate the relevant mechanisms that will trigger tendon regeneration. On the contrary, aberrant mechanical loading alters the anabolic processes in tendons, resulting in the differentiation of tendon stem cells into nontenocytes, such as osteocytes, which may lead to the development of degenerative tendinopathy [48–50]. Our previous studies demonstrated that the mechanical cyclic loading protocol used in this study, i.e., 8% strain at 1 Hz frequency produced the most optimal level of tenogenic differentiation for BM-MSCs [38]. The assumption made in this study was that AD-MSCs would respond in a similar manner to BM-MSCs when subjected to the same loading regimes. There may be a flaw in this assumption as the maximum tenogenic potential of AD-MSCs using a different regime was neither really explored nor revealed. Nevertheless, the present method did demonstrate a comparable potency to BM-MSCs, which was sufficient to answer our hypothesis.

In the present study, gene expression were the main indicators demonstrating the effects of stretching and that using *COL1*, *COL3*, *DCN*, *TNC*, and *BGN* as markers of tenogenic differentiation, and the experiments proved our hypothesis that stretching would indeed promote tenogenic expression. Such assumptions were in fact demonstrated previously in our prior report [38] and other studies such as those reported by Youngstrom et al. [51]. The choice of the panel of gene expression being investigated was reasonable having understood that these would lead to the protein expression which contributes to the matrix formation of tendon formation and repair. It is known that collagen type I is the primary matrix component of mature tendon/ligament, albeit not being exclusive to tendon tissue alone. Other matrix molecules including collagen types III, XII, and XIV; elastin; and proteoglycans may reflect the contents of tendon, albeit is in lower volume and is varied. Nevertheless, these are not specific to tendon as well. However, when these are considered holistically, these markers inherently provide sufficient indications of the ongoing repair process. In our experiments, we have demonstrated an increase in the synthesis of collagen types I and III at gene transcriptional levels, of which in tendons, this ratio has been used as an indicator of preferable tendon tissue repair outcomes [52, 53]. Such notion is supported further when several studies have indicated that collagen type III is increased during process of mechanotransduction process [54–56], which is apt for the present experimental purposes. In general, the presence of collagen type I is important to resist mechanical loading whilst collagen type III is found to be involved in the early stages of tendon and ligament healing [53]. Therefore it is understandable that the ratio of collagen type III to collagen type I is being used in some studies, and that an increase in this ratio indicates tissues are undergoing the early stages of tendon healing [52, 57–59]. The use of other protein expressing gene markers, such as Decorin, provides further supporting evidence for the anticipated repair outcomes since this protein is the predominant proteoglycan component located in the tensile region of tendons which implicated in lateral fibril growth [60]. This fibre has been shown to correlate with size and density of collagen fibrils, and thus of the mechanical strength of tendon tissue itself [61]. Likewise, *BGN* is important for directing assembly of collagens [62], in addition to be essential in the maintenance of the putative tendon stem cell niche [63].

In considering the larger view of things, the relationship of *COL*, *DCN*, and *BGN* in our current studies appears overarching and provides a good and comprehensive indicator of the potential healing capacity of MSCs. In addition to expressing specific tendon related proteins, mechanical stimulation also triggers specific signalling pathways that lead to transcription of the regulatory genes of resident progenitor cells and in introducing MSCs into damaged tendon these cells, towards the activation of tenogenesis differentiation pathway. The mitogen-activated protein kinase (MAPK) pathway was found to be up-regulated in MSCs exposed to cyclic tensile strain, suggesting it as an important mechanotransductive pathway in MSCs differentiation [64, 65]. Another study of Kearney et al. found ERK and p38 to be involved in cyclic tension mechanotransduction, and stretch-activated cation channels are implicated to mediate collagen I gene expression [66]. Whilst there have been previous publications describing the effects of mechanical straining on MSCs differentiation, these mainly focuses on the differentiation of cells into smooth muscle cells, chondrocytes, and osteoblasts [26, 67, 68]. Like previous reports, our study demonstrated that AD-MSCs had good multilineage differentiation capacity and good cellular proliferation that was comparable to bone marrow [69, 70]. Nevertheless, it became apparent from our gene expression that uniaxial cyclical tensile loading on these two cells sources suppressed adipogenesis, chondrogenesis, and osteogenesis and instead strongly promoted tenogenesis. This appears to complement the results from our previous study involving human bone marrow MSCs [28]. Interestingly though, whilst prior report had mentioned that AD-MSCs produced superior mechanotransductive responses BM-MSCs when subjected to mechanical stimulation, our study was not able to establish this [41].

Whilst the study design was sufficiently robust, there were notable limitations to the present study. Firstly, inherent to any *in vitro* study design, biological variations that may occur due to the multiple donors for cell sources is unavoidable and may have influenced the study results. In order to reduce this, in most cases, we did our best to obtain both bone marrow and adipose derived-MSCs from similar individuals. This increased the likelihood to produce desirable observable related changes but does not address the variability of different individuals. To reduce the variation effect, a large sample size involving large population would be necessary, which in a laboratory experiment would be logistically and economically prohibitive. We accept this limitation in any *in vitro* experiment and interpreted the results accordingly to merely demonstrate the potential clinical efficacy prior to validating our results into a larger scale preclinical and/or clinical studies. Hence, we suggest a more robust and deeper investigation in the near future that would be necessary verify the findings of the present study before any further clinical implications can be made. Secondly, our preliminary study has shown that the size of cell culture chamber/device provide limited space for cellular expansion and results in maximal size expansion or confluency when experiments were conducted up to 48 to a maximum of 72 hours. In our previous study, we were able to demonstrate that the tenogenic differentiation of BM-MSCs could be triggered as earlier as 24 hours and enhanced phenotypic expression at 48 hours using the proposed mechanical strain and frequency. This would have been sufficient for the needs for the present experiments. To further extend the study beyond this would result in aberrant results as cells in culture would undergo proliferative contact inhibition. This was the rationale to limit the experiment to 48 hours. Moreover, considering that the continuous mechanical stimulation is a form of accelerated outcome stimuli, any results from this experiment would have been observed within this time period.

## 5. Conclusions

The present study demonstrates that mechanical stretching at 1 Hz and 8% strain did not promote cellular promotion but enhance tenogenic differentiation and protein expression for both AD-MSCs and BM-MSCs equally, suggesting that both cell sources are equally suitable in treating damaged tendon.

## Figures and Tables

**Table 1:** The genes of interest were determined in this study.

Related marker	Gene name	Abbreviation	Ref sequence	Catalog number
Tendon lineage	Collagen type I, *α*1	*COL1A1*	NM_000088	PPH01299F
	Collagen type III, *α*1	*COL3A1*	NM_000090	PPH00439F
	Decorin	*DCN*	NM_001920	PPH01900A
	Tenascin C	*TNC*	NM_002160	PPH02442A
	Biglycan	*BGN*	NM_001711	PPH01899A

Other mesenchyme lineage	Runt-related transcription factor 2	*RUNX2*	NM_001015051	PPH01897C
	SRY-(sex determining region Y-) box 9	*SOX9*	NM_000346	PPH02125A
	Peroxisome proliferative activated receptor, gamma	*PPARG*	NM_005037	PPH02291G
	Transgelin	*TAGLN*	NM_001001522	PPH19531F

Housekeeping gene	Phosphoglycerate kinase 1	*PGK1*	NM_000291	PPH02049A
	Hypoxanthine phosphoribosyltransferase 1	*HPRT1*	NM_000194	PPH01018C

**Table 2:** Flow-cytometric analysis of expanded passaged-2 AD-MSCs and BM-MSCs.

Surface protein	% positive AD-MSCs	% positive BM-MSCs
*Positive hMSCs markers*		
CD73	92.5	94.8
CD44	99.1	98.8
CD90	100.0	100.0
CD105	96.9	97.0

*Negative hMSCs markers*		
CD14	0.3	1.2
CD34	5.5	2.2
CD45	0.3	0.7
HLA-DR	1.2	2.4

## Data Availability

The data used to support the findings of this study are included within the article.
